# Dendritic location of dystrophic neurites in FTLD‐TDP type C with annexinopathy

**DOI:** 10.1111/bpa.70032

**Published:** 2025-07-23

**Authors:** Allegra Kawles, Antonia Zouridakis, Caroline Nelson, Rachel Keszycki, Grace Minogue, Alyssa Macomber, Pouya Jamshidi, Rudolph J. Castellani, Changiz Geula, Tamar Gefen, M‐Marsel Mesulam

**Affiliations:** ^1^ Mesulam Institute for Cognitive Neurology and Alzheimer's Disease Northwestern University Feinberg School of Medicine Chicago Illinois USA; ^2^ Department of Psychiatry and Behavioral Sciences Northwestern University Feinberg School of Medicine Chicago Illinois USA; ^3^ Department of Pathology Northwestern University Feinberg School of Medicine Chicago Illinois USA; ^4^ Department of Cell and Developmental Biology Northwestern University Feinberg School of Medicine Chicago Illinois USA; ^5^ Department of Neurology Northwestern University Feinberg School of Medicine Chicago Illinois USA

**Keywords:** dystrophic neurites, FTLD‐TDP, FTLD‐TDP‐C, TDP type C, TDP‐43

## Abstract

The type C variant (TDP‐C) of FTLD‐TDP exhibits unique features, not shared by types A and B, namely the invariable and frequently asymmetric predilection for the anterior temporal lobes (ATL). Depending on the direction of hemispheric asymmetry, the associated clinical features include word comprehension impairment, associative agnosia, and behavioral abnormalities. Current research on TDP‐C aims to explore the factors that underlie the selective targeting of the ATL and, more specifically, the cellular details that undermine the behavioral and cognitive functions of this region. Abnormal TDP‐C neurites have recently been shown to represent heterodimers with annexin A11 (ANXA11). This feature, not shared by TDP‐A or ‐B, may explain the unique predilection of TDP‐C for the ATL. To further explore the subcellular distribution of the pathology, paraffin‐embedded sections were stained using fluorescent antibodies for the dendritic marker MAP2 and phosphorylated TDP‐43 (pTDP) or ANXA11. Results indicated that approximately half of pTDP/ANXA11 neurites co‐localized with MAP2. The actual overlap during life may be much higher but decreased at autopsy through dendritic loss due to prolonged neurodegeneration. The potentially selective and progressive dendritic pathology of TDP‐C, quite unique among neurodegenerative entities, may underlie the distinctive perturbation of cortical integrative computations.

## Graphical abstract

The type C variant of FTLD‐TDP (TDP‐C) is defined by long dystrophic neurites that are immunopositive for both phosphorylated TDP and annexin A11 (ANXA11). Kawles et al. are the first paper to systematically show that these neurites are likely of dendritic origin.

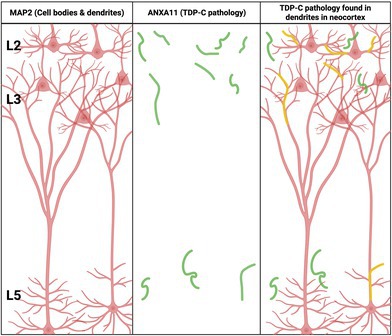

## 1 INTRODUCTION

Transactive response DNA‐binding protein 43‐kDa, or TDP‐43, is an RNA/DNA‐binding protein predominantly localized in the nucleus in non‐pathologic states. In its pathologic state, TDP‐43 mislocalizes from the nucleus, becoming truncated and hyperphosphorylated [1, 2]. Frontotemporal lobar degeneration due to TDP‐43 proteinopathy (FTLD‐TDP) is a neurodegenerative disease where aggregations of pathologic hyperphosphorylated TDP (pTDP) are primarily found in frontal and temporal brain regions. FTLD‐TDP is further classified into three main pathologic substrates (types A, B, and C), which are differentially defined by the presence and localization of intranuclear, cytoplasmic, or neuritic pTDP inclusions [3]. TDP type A is characterized by neuronal cytoplasmic inclusions and short neurites in upper cortical layers and can be associated with *GRN* mutations. TDP type B shows compact cytoplasmic inclusions with few dystrophic neurites across cortical layers and can be associated with C9orf72 mutations. TDP type C (TDP‐C) is pathologically distinct from these subtypes due to the presence of pathognomonic long, thick neurites found predominantly in superficial cortical layers. TDP‐C is also the only major FTLD‐TDP subtype with no convincing association with disease‐causing mutations.

Recent studies using immunohistochemistry [4] and cryogenic electron microscopy [5] reveal that TDP‐C neurites contain a heteromeric amyloid filament of pTDP and annexin A11 (ANXA11), a feature absent in TDP‐A and ‐B. ANXA11 is a calcium‐dependent phospholipid binding protein typically found in the cytoplasm. Its function is a molecular tether between RNA stress granules and lysosomes that are “hitchhiking” down microtubules to be delivered to other parts of the neuron [6, 7, 8]. Interestingly, non‐pathologic TDP‐43 is often among the proteins found in the RNA granules that ANXA11 assists [9]. Mutations in ANXA11 have previously been implicated in amyotrophic lateral sclerosis, which is typically associated with FTLD‐TDP type B [10]. Recently, ANXA11 variants have been implicated in TDP‐C [4, 11] and other associated dementia syndromes [12]. Little is known regarding the interaction of pTDP and ANXA11 in TDP‐C, other than that their dimerization appears to be a fundamental feature of TDP‐C pathogenesis.

Beyond its unique pathological features, TDP‐C also shows a rare affinity for the anterior temporal lobe (ATL). In a recent study, we found that of the participants that have come to autopsy at the Northwestern University Alzheimer's Disease Research Center (NU‐ADRC), those diagnosed with TDP‐C showed an uncommonly uniform atrophy pattern such that the ATL was always the principal site of atrophy [13]. All of these individuals presented clinically either with the semantic variant of primary progressive aphasia (svPPA) or behavioral variant frontotemporal dementia (bvFTD), reflecting clinicoanatomic correlations that show object naming and word comprehension to be dependent on the language‐dominant (usually left) ATL, whereas behavioral control and non‐verbal object recognition display a more bilateral representation with a rightward ATL bias [14, 15]. The mechanisms underlying the profound behavioral and cognitive impairments caused by ATL neurodegeneration in TDP‐C remain unclear, particularly given that similar outcomes are not observed following resection of this region (or more) for the treatment of temporal epilepsy [16]. Furthermore, other neurodegenerative pathologies causing severe ATL atrophy do not reliably lead to such pronounced, defined deficits [17, 18]. This suggests that in addition to the location of the damage, specific cellular details of TDP‐C pathology also play a critical role in the emergence of the clinical syndrome.

The presence of long neurites, absence of associated disease‐causing mutations, and the co‐assembly of pTDP and ANXA11 distinguish TDP‐C as a pathologically distinct form of TDP‐43 proteinopathy. Given the morphology of neurites and their absence in the white matter, we hypothesized that TDP‐C neurites are found in the dendrites of neurons. To date, one paper has suggested that TDP‐immunopositive neurites are of dendritic origin [19]. In this study, we used fluorescent double immunohistochemistry to determine co‐localization of dystrophic pTDP/ANXA11 neurites with dendritic processes identified by MAP2. Our findings reinforce the dendritic origin of TDP‐C neurites and offer evidence that the overlap may be surprisingly high during the patient's lifetime. This overlap at the subcellular level leads to important questions about the computational mechanisms that link TDP‐C neurodegeneration in ATL to the severe disruption of cognitive and behavioral integration.

## 2 METHODS

### 2.1 Case characteristics

Seven cases with a clinical diagnosis of svPPA and autopsy‐confirmed TDP‐C as the primary pathologic diagnosis were identified from the NIA‐funded Alzheimer's Disease Research Center Brain Bank housed within the Mesulam Institute for Cognitive Neurology and Alzheimer's Disease at Northwestern University's Feinberg School of Medicine. All cases were co‐enrolled in the Northwestern PPA Program. Written informed consent was obtained from all participants who committed to brain donation. PPA clinical diagnosis was determined by a behavioral neurologist and required a clinical history of progressive language impairment unaccompanied by consequential decline in other cognitive domains within the initial stages of the disease [20, 21]. Further classification into PPA subtypes was based on retrospective chart review, guided by the criteria of Gorno‐Tempini et al. (2011) and Mesulam et al. (2009) [22, 23]. Five of seven participants were female. The mean age at symptom onset was 55.29 years (SD = 4.72), and the mean age at death was 68.29 years (SD = 4.82). Disease duration ranged from 11 to 14 years, in line with published data that PPA due to TDP‐C shows an elongated disease duration [24]. Six of seven participants were right‐handed; all cases showed left‐predominant atrophy in vivo and at autopsy, indicating a left‐lateralized language network. No cases had a known mutation associated with Alzheimer's Disease (AD) or FTLD. See Table 1 for demographic and participant information.

**TABLE 1:** Participant information.

Case	Sex	Clinical Dx	Age of onset (years)	Age at death (years)	PMI (hrs)	Brain weight (g)	Handedness	Secondary pathologies
P1	F	svPPA	51	65	18	1000	R	Moderate vascular disease; ADNC Low (A2, B0, C2)
P2	F	svPPA	58	71	38	1038	R	ADNC low (A0, B1, C0)
P3	M	svPPA	60	73	6	1370	L	ADNC low (A1, B0, C0); moderate vascular disease
P4	F	svPPA	50	64	34	858	R	Moderate vascular disease
P5	F	svPPA	50	61	UNK	840	R	ADNC low (A1, B0, C0); mild vascular disease; bilateral hippocampal sclerosis
P6	F	svPPA	60	72	32	1056	R	ADNC low (A3, B1, C2); mild vascular disease; pigment spheroid degeneration
P7	M	svPPA	58	72	3	957	R	Moderate vascular disease

Abbreviations: ADNC, Alzheimer's disease neuropathologic change (A = Thal phase, B = Braak stage; C = CERAD score); F, female; L, left; M, male; PMI, postmortem interval; R, right; svPPA, semantic variant primary progressive aphasia; UNK, unknown.

### 2.2 Neuropathological evaluation and histological preparation

**FIGURE 1:**
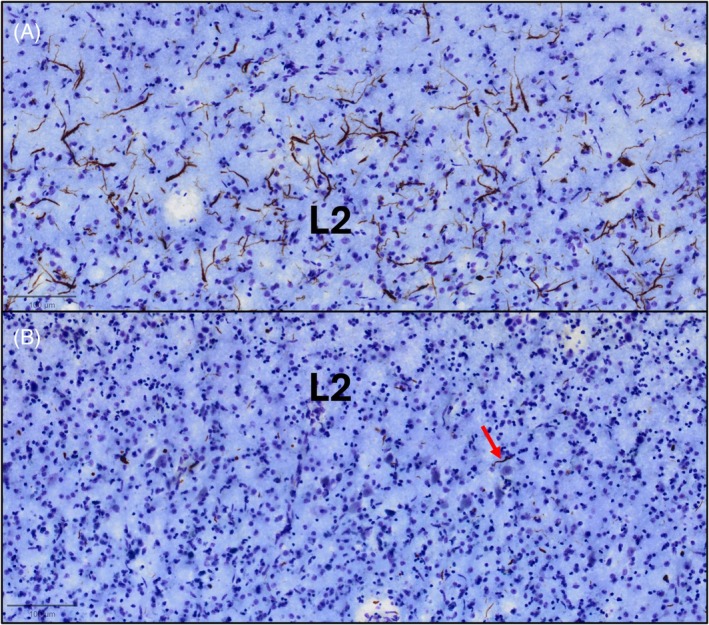
Distribution of neurites in right IFG and ATL. (A) and (B) taken from a 65‐year‐old female with a 16‐year history of svPPA. Postmortem brain tissue was sectioned at 40 μm thickness and immunostained with phosphorylated TDP and counterstained with cresyl violet to visualize TDP‐C neurites and neurons, respectively. Upper cortical layer II (L2) is noted; both images are oriented with pial surface at the top of the figure. (A) depicts right Brodmann area (BA) 44, or pars opercularis of IFG, where there are plenty neurites in upper cortical layers of various morphologies. (B) depicts right ATL in the same case. Here, there are few neurites (red arrow), and the tissue is gliotic with very few underlying neurons. While the ATL is an important region in TDP‐C, analyses of neuronal processes and inclusions would be extremely difficult. Bar in (A) and (B) indicates 100 μm.

Mean postmortem interval (PMI) was 21.8 h, and mean brain weight was 1017 grams. Following autopsy, the cerebral hemispheres were separated in the midsagittal plane, cut into 2‐ to 3‐cm coronal slabs, fixed in formalin or 4% paraformaldehyde for 36 h, taken through sucrose gradients (10%–40%) for cryoprotection, and stored in 40% sucrose with 0.02% sodium azide at 4°C. The pathologic diagnosis of FTLD and specification of its variants was rendered by a neuropathologist (RJC) using published consensus criteria of the Consortium for FTLD [25]. No cases had an extent of AD pathology (ADNC) above “low” according to Hyman et al. and Montine et al. [26, 27], with Braak staging ranging from 0 to 1. No cases had co‐morbid alpha‐synucleinopathy pathology. For all cases except P4, samples were taken from bilateral inferior frontal gyrus (IFG; Brodmann Area 44), embedded in paraffin, and cut into three consecutive 5 μm‐thick sections per region per case; P4 only had right IFG available. Bilateral IFG was selected given prior research from our group that implicates it as a region with a relatively preserved neuronal population with at least moderate pathologic inclusion density [28]. The ATL, which is a central region of interest in the study of TDP‐C, was not analyzed in this study because (1) its vulnerability to TDP‐C leaves the tissue gliotic and frail by the time cases come to autopsy, leading to limited analysis of neuronal integrity, and (2) there are few neurites to analyze postmortem (Figure 1). This observation is further described in Kawles et al., 2022 [28].

### 2.3 Immunofluorescent staining and digital analysis

Sections were double‐stained immunohistochemically in parallel with anti‐microtubule‐associated protein 2 (MAP2) antibody (Synaptic Systems, polyclonal guinea pig, 1:1500; Cat No. 188004) and with a pTDP‐43 antibody (pSer409/410; CosmoBio, polyclonal rabbit, 1:1000; Cat No. CAC‐TIP‐PTD‐P03) or with an ANXA11 antibody (ProteinTech, polyclonal rabbit, 1:100; Cat No. 10479‐2‐AP). For double immunofluorescent staining with pTDP and ANXA11, a mouse monoclonal antibody was used (pSer409/410; CosmoBio, monoclonal mouse, 1:1000; Cat No. CAC‐TIP‐PTD‐M01A). MAP2 antibody visualizes neuronal perikarya and dendrites, and phosphorylated TDP and ANXA11 visualize the long neurites in TDP‐C. Antibodies were visualized with fluorescently labeled secondary antibodies (Invitrogen Alexa Fluor™ 488 (Cat No. A‐110008) and Alexa Fluor™ 647 (Cat No. A‐21450 or A‐21235)). Sections were coverslipped with DAPI‐containing mounting media to visualize nuclei (ProLong™ Diamond Antifade Mountant; Cat No. P36962). A digital image of each slide was obtained at 20× magnification using the Olympus VS200 Slide Scanner, using FITC and Cy5 channels to visualize targets tagged with the Alexa Fluor™ 488 and 647, respectively. Slides were analyzed using QuPath software (v.0.5.1).

Briefly, a section of the gray matter was traced from the cortical surface to the white matter, and a 500 × 500 μm grid was overlaid on the image. A different section of the gray matter was analyzed for each slide. The FITC and DAPI channels were selected for viewing, thereby visualizing only DAPI with pTDP or ANXA11 and “hiding” the Cy5 red channel that would visualize MAP2. Using the ellipse annotation tool, 3–5 neurites were then randomly selected per grid square. The Cy5 channel was then selected, revealing MAP2 immunostaining. Each previously selected neurite was reviewed for co‐localization with MAP2, and an annotation was marked if co‐localization was present. Percentage of neurites showing co‐localization was calculated for each slide using the ratio of marked to total annotations. Ratios were averaged across the three slides per region. A student's *t*‐test compared the percentage of co‐localized neurites in the right and left IFG.

## 3 RESULTS

### 3.1 Co‐localization of pTDP with dendritic marker MAP2

Co‐localization of pTDP with MAP2 protein ranged from 40% to 80%. Co‐localization was observed with both thick neurites and thinner, wispy neurites. In some instances of co‐localization, MAP2 immunostaining appeared ballooned and diffuse, similar to dendritic swelling. pTDP co‐localized both perpendicularly and parallelly oriented to the pial surface, potentially representing both basal and apical dendrites.

### 3.2 Confirmation of co‐localization of pTDP with ANXA11

**FIGURE 2:**
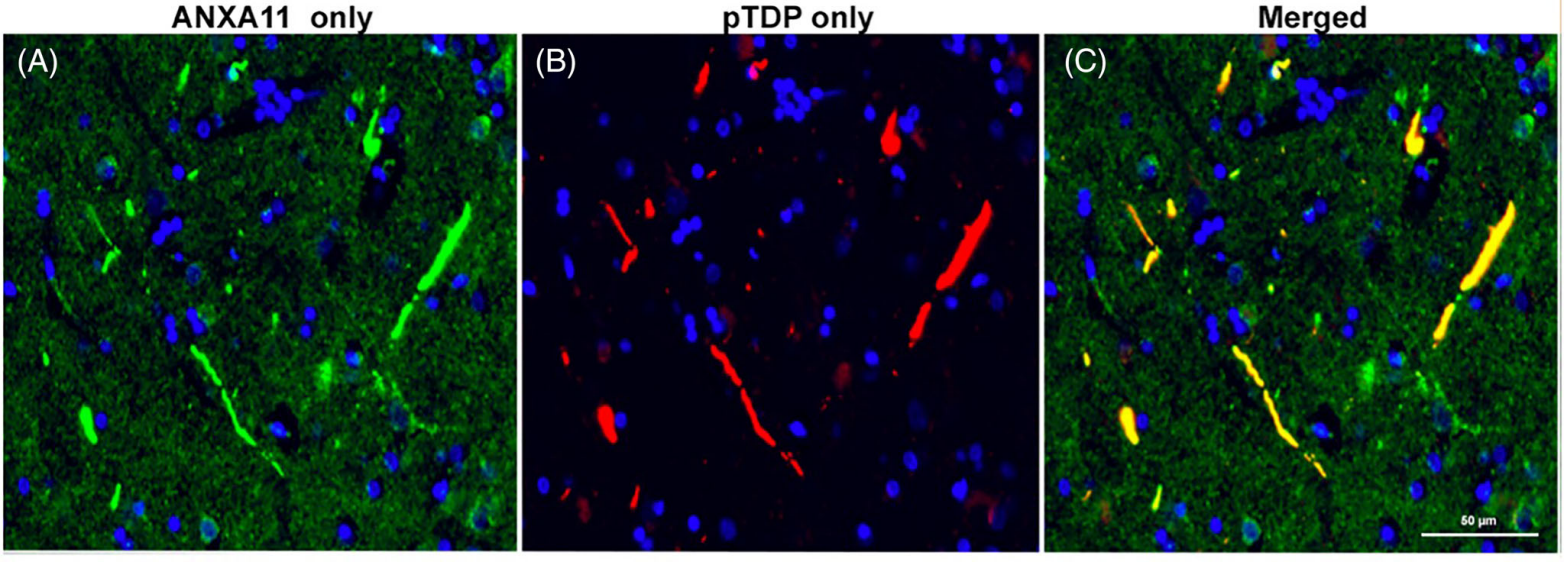
(A–C) Nearly total co‐localization of pTDP (red) with ANXA11 (green) in TDP‐C neurites in right inferior frontal gyrus (IFG), consistent with prior research. Image taken from right IFG of P4, a 64‐year‐old female with a 14‐year history of svPPA. Image is oriented so that the pial surface would be found at the top of figure. Both characteristic long, thick neurites as well as shorter neurites show co‐localization with ANXA11. In addition, there were instances of smaller neurites that were ANXA11‐positive and pTDP‐negative, which may represent pathologic ANXA11‐only neurites, or healthy, typical ANXA11 that may be present in dendritic processes. Bar in (C) indicates 50 μm and applies to (A–C). Blue represents nuclei stained by DAPI.

Prior studies have determined the co‐localization of pTDP with ANXA11 in TDP‐C using cryogenic electron microscopy, fluorescent and brightfield immunohistochemistry, and western blotting [4, 5]. As confirmation, we analyzed fluorescent immunohistochemistry to determine the level of co‐localization between ANXA11 and pTDP in TDP‐C. Analyses revealed 100% co‐localization of pTDP‐immunopositive neurites with ANXA11 protein (Figure 2). Morphologically, ANXA11 virtually mirrored the morphology of neurites typically seen in TDP‐C. While there were no instances of pTDP without ANXA11, there were instances of ANXA11 without pTDP (ANXA11‐positive, pTDP‐negative neurites); specifically, ANXA11 immunopositivity often appeared to extend past the pTDP in the neurite, such that the ANXA11‐immunopositive neuritic inclusion was longer than the pTDP‐immunopositive neuritic inclusion. ANXA11 immunopositivity was also noted in the cell body.

### 3.3 ANXA11 as a proxy for pTDP neurites and its co‐localization with MAP2

**FIGURE 3:**
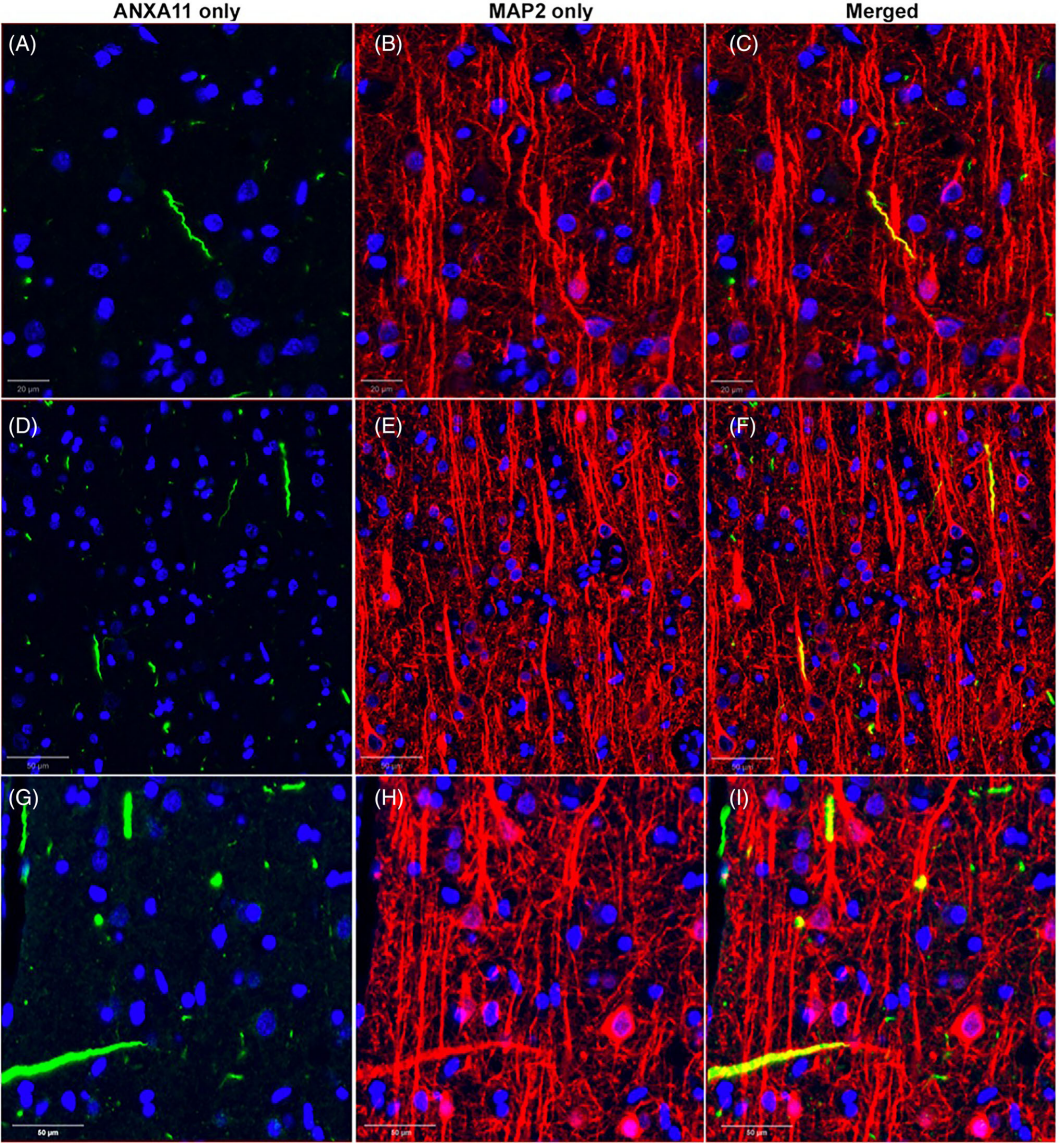
Co‐localization of ANXA11 with MAP2 protein suggests dendritic origin of neurites. (A–I) are taken from right IFG sections, and all are oriented with pial surface at top of figure. Co‐localizing neurites are both thick and rectilinear, and thin and wispy. Vertical neurites seen in (D and G) are likely reminiscent of apical dendrites. A may represent an apical dendrite that is undergoing degeneration, changing its morphology and orientation. Thick, horizontal neurites in (G) may be basal dendrites or branching apical dendrites. (A–F) images were taken from P5, a 61‐year‐old female with an 11‐year history of svPPA. (G–I) images were taken from P1, a 65‐year‐old female with a 14‐year history of svPPA. Blue represents nuclei stained by DAPI.

Double immunofluorescent staining was repeated with ANXA11 and MAP2 in bilateral IFG (Figure 3). Using similar methods, we found that approximately 60% of ANXA11‐immunopositive neurites co‐localized with MAP2 across cases. There was no statistically significant difference between the percent of co‐localized neurites in right and left IFG (right IFG M = 62.1%, SD = 12.5; left IFG M = 61.4%, SD = 8.4). ANXA11/MAP2 double immunofluorescent staining was clearer and more consistent; that is, neurites appeared more whole and uniformly immunopositive compared to prior double staining with pTDP. Co‐localizing neurites were again found both perpendicularly and parallelly oriented to the pial surface, though more perpendicular instances of co‐localization were noted with ANXA11.

## 4 DISCUSSION

The present study found that a surprisingly high proportion of these TDP‐C neurites overlap dendritic profiles identified by MAP2 immunoreactivity. Many of the MAP2‐positive pTDP/ANXA11 neurites were vertically oriented and found primarily in cortical layers I, II, and IIIa, with fewer in layers V and VI. Morphologically, these structures appear to be dendritic components of cortical pyramidal neurons, which have large, apical dendrites that ascend vertically across cortical layers and ramify into thinner, horizontal branches. MAP2‐positive pTDP/ANXA11 neurites in deeper cortical layers (i.e., V and VI) may be remnants of basal dendrites, which extend from the base of pyramidal neurons [29]. pTDP/ANXA11 neurites are almost entirely absent from the white matter, further reinforcing their dendritic rather than axonal identity. Interestingly, some neurites did not display a typical dendritic shape but still co‐localized with MAP2. These neurites, which appeared as complex, tangled structures, may represent dendrites that have undergone pathological dysmorphic alterations.

Dendritic dysfunction and degeneration are key features of many neurodegenerative and psychiatric diseases [30, 31]. For example, Alzheimer's disease, amyotrophic lateral sclerosis, and Parkinson's disease have been shown to extensively alter both dendritic spines and arborization (see reviews in Herms and Dorotskar [32]; Luebke et al. [33]). A recent study found that levels of synaptic proteins, and particularly the dendritic spinule protein spinophilin, were decreased in FTLD‐TDP, predominantly types A and B, compared to age‐matched controls. Aggregates of these synaptic proteins were found within microglia, suggesting aberrant synaptic pruning as a pathological feature of TDP‐A and ‐B [34]. However, in the absence of the colocalization we see in TDP‐C, these effects on dendrites in other neurodegenerative diseases, including TDP‐A and B, are likely to reflect secondary consequences of upstream neuropathologic processes. The closest analog to the predominantly dendritic localization of TDP‐C neurites is the neuropil threads found in Alzheimer's disease neuropathology. Braak and Braak (1988) determined the dendritic origin of neuropil threads, noting that these threads were only found in tangle‐bearing neurons [35], making them markers of downstream neurodegeneration rather than an inciting cause [36].

The possibility that TDP‐C preferentially targets the dendrites of large pyramidal neurons is particularly significant, as these neurons—and specifically their arbors in layer II—play a strategic role in the feedforward‐feedback interactions that underlie integrative cortical function [37]. These considerations are particularly relevant to the ATL, which serves as the downstream convergence point for multiple processing streams that must communicate in complex and varied ways [38]. For example, in object naming and word knowledge, which are disrupted in svPPA, the left ATL is essential for linking a word to the perception of its corresponding object, as well as for integrating related pieces of semantic information. Consequently, the ATL is likely to support an unusually high load of plasticity to enable the continued acquisition of new associations and their incorporation into semantic networks. Indeed, the ATL is among the deepest synaptic levels in sensory‐fugal pathways such that the effects of its degeneration, even at the dendritic level, are felt across other modality‐specific regions [39]. These processes require high levels of synaptic sculpting, which may render the ATL, specifically its dendrites, preferentially susceptible to metabolic stress and abnormal proteostasis. In contrast to the profound neurointegrative dysfunction caused by TDP‐C, other entities that are even more destructive to the ATL, such as neurosurgical ablations and certain cases of Pick's disease, have a much more muted and transient impact on cognition and behavior [16, 17, 18]. The difference may reflect the selective dendritic disruption in TDP‐C, which is likely to cause network perturbations more destructive to network computations than complete tissue loss. Further, selective dendritic disruption may in part explain the particularly long disease duration of TDP‐C, where patients can often functionally compensate for word knowledge deficits for many years [24, 38].

The overlap between pTDP/ANXA11 neurites and dendritic markers was not complete. Methodological limitations, such as antibody occlusion, may have prevented the more extensive co‐localization of neuritic deposits with cytoskeletal proteins. Dendrites, in particular, are challenging to visualize and may not have been fully revealed by MAP2 immunohistochemistry. Indeed, while MAP2 immunostaining is often used to visualize full neuronal processes, MAP2 mRNA is highest in proximal dendrites [40]. The use of thin, 5‐μm thick sections may have also limited visualization, particularly for dendrites. Furthermore, by the time of autopsy, which occurs more than a decade after disease onset, many dendrites would have been lost and many pTDP/ANXA11 neurites would have been resorbed [28]. According to this line of reasoning, the overlap between TDP‐C neurites and dendrites may be much higher during the initial years of disease than the 60% shown at post‐mortem by this study. Additional limitations include the small sample size (*N* = 7) and disproportionate male‐to‐female ratio. Future studies will aim to further confirm these exciting preliminary results through analyses of dendritic integrity across anatomic regions of TDP‐C.

## AUTHOR CONTRIBUTIONS

AK, CG, TG, and MMM conceived and designed the project. PJ and RJC prepared postmortem samples for analyses, including the determination of appropriate neuropathologic diagnoses. AK, AZ, CN, RK, GM, and AM contributed to data collection. AK, AZ, and CN analyzed data. AK and MMM drafted the initial manuscript and figures. CG, TG, and MMM provided supervision. All authors participated in data and manuscript review and editing and approved the final manuscript.

## CONFLICT OF INTEREST STATEMENT

The authors declare no conflicts of interest.

## ETHICS STATEMENT

All procedures performed in studies involving human participants were in accordance with the ethical standards of The Northwestern University Internal Review Board and with the 1964 Helsinki declaration and its later amendments or comparable ethical standards. This article does not contain any studies with animals performed by any of the authors. Written informed consent was obtained from all individual participants included in the study.

## Data Availability

Data are available to investigators upon reasonable request and approval from the Northwestern Alzheimer's Disease Research Center. To learn more about the collaborative process and instructions to request data, see: https://www.brain.northwestern.edu/scientists‐students/collaborative‐request.html.
